# Opportunities for optimization of in-field water harvesting to cope with changing climate in semi-arid smallholder farming areas of Zimbabwe

**DOI:** 10.1186/2193-1801-2-100

**Published:** 2013-03-11

**Authors:** George Nyamadzawo, Menas Wuta, Justice Nyamangara, Douglas Gumbo

**Affiliations:** 1 Department of Environmental Sciences, Bindura University of Science Education, Box 1020, Bindura, Zimbabwe; 2 Department of Soil Science and Agricultural Engineering, University of Zimbabwe, Mount Pleasant, Box MP167, Harare, Zimbabwe; 3 ICRISAT Bulawayo, Box 776, Bulawayo, Zimbabwe

**Keywords:** Water conservation, In-field water harvesting, Semi arid, Climate change, Adaptation, Tied contours

## Abstract

Climate change has resulted in increased vulnerability of smallholder farmers in marginal areas of Zimbabwe where there is limited capacity to adapt to changing climate. One approach that has been used to adapt to changing climate is in-field water harvesting for improved crop yields in the semi- arid regions of Zimbabwe. This review analyses the history of soil and water conservation in Zimbabwe, efforts of improving water harvesting in the post independence era, farmer driven innovations, water harvesting technologies from other regions, and future directions of water harvesting in semi arid marginal areas. From this review it was observed that the blanket recommendations that were made on the early conservation method were not suitable for marginal areas as they resulted in increased losses of the much needed water. In the late 1960 and 70s’, soil and water conservation efforts was a victim of the political environment and this resulted in poor uptake. Most of the water harvesting innovations which were promoted in the 1990s’ and some farmer driven innovations improved crop yields in marginal areas but were poorly taken up by farmers because they are labour intensive as the structures should be made annually. To address the challenges of labour shortages, the use of permanent in-field water harvesting technologies are an option. There is also need to identify ways for promoting water harvesting techniques that have been proven to work and to explore farmer-led knowledge sharing platforms for scaling up proven technologies.

## Introduction

Climate change has resulted in increased food insecurity in the smallholder farming sector in Africa and Zimbabwe has not been spared. The most vulnerable people are the resource poor farmers, the elderly, women, children, and women and child-headed households because they have limited adaptive capacity. Marginal areas which receive low rainfall are also vulnerable to climate change because in most cases the rainfall is not adequate to sustain crop production. Semi-arid regions which receive the lowest rainfall in Zimbabwe also have the least reliable distribution varying from 20% in the north to 45% in the south (Department of Meterological Services 1981; Bratton 1987).

Recurrent droughts have often resulted in severe crop damage, decreased livestock production and widespread food shortages and the most severe impacts of droughts are felt in countries with agro-based economies. In most countries with limited capacities to adapt to climate change, recurring droughts have often led to severe food shortages. In addition, as crop yields decline with changing climate, pressure to cultivate unsuitable land will rise. This is a major challenge, as productivity from land and water in many tropical regions will decline due to land degradation (UNEP 1992).

The global food challenge is huge and it will be a challenge to feed the additional 3 billion people by 2050 (Conway 1997). About 95% of this population growth will occur in developing countries and this is a developmental challenge as most of their economies are agricultural based. The majority, or two thirds of the poorest people in the world are found among the 1.1 billion farmers who make their living from agriculture (Rockström 2002). In Zimbabwe which is one example of an agro-based economy, households have been facing perennial drought related food shortages and they have been surviving on donor funded food aid programmes. In 2011/2012 season, the estimated number of people requiring food aid stood at 1.7 million (World Food Program of the UN 2012; The Zimbabwe Independent 2012; The Herald 2012), while in 2010/2011, it was estimated that 1.4 million people required food aid in Zimbabwe.

Climate change models have projected a decrease in rainfall in southern Africa (New et al. 2006), and research has already shown the same trends (Nyagumbo et al. 2009a). Decreasing rainfall in semi arid regions implies worsening food shortages if the current farming practices do not improve. Decreasing rainfall is a challenge as most of the agricultural systems of southern Africa are predominantly rain-fed as irrigation systems are not well developed (Camberlin et al. 2009). Thus, we are faced with huge water for food challenge, and the focus should be on upgrading rain-fed smallholder farming in tropical environments characterized by frequent droughts and mid-season dry spells.

In addition, most of the rainfall received in semi arid regions is lost as runoff, and very little water is harvested for plant growth or future use. In Zimbabwe, losses >50% of received rainfall have been reported (Nyamadzawo et al. 2012). High levels of runoff losses in smallholder farming areas do not only limit water availability, but are also an erosion hazard and cause nutrient losses (Elwell and Stocking 1988). Researchers studying soil erosion are in agreement that parts of Zimbabwe’s smallholder areas face serious erosion problems (e.g. Elwell and Stocking 1988; Whitlow 1988). Whitlow and Campbell (1989) reported that over 25% of the smallholder areas are severely eroded and this has been cited as one of the major causes of poor yields.

In Zimbabwe rainfall is the principal water resource for agriculture. However, the rainfall exhibits a high degree of inter-annual variability. Droughts of several years duration, such as that which occurred from 1981 to 1984 have been recorded in southern Africa (Tyson 1986) and from 1959 to 2002; Zimbabwe experienced 15 droughts occurring on average, every 2 to 3 years (World Bank 2009). In some semi-arid smallholder farming regions the rainfall patterns and distribution have changed and there has been an increase in the average duration of intra-seasonal dry spells (New et al. 2006). All this has resulted in perennial food shortages due to insufficient rainfall which causes poor yields. It is becoming increasingly clear that to face the food challenge over the coming 50 years, combined efforts of developing climate smart rainfed and irrigated agriculture will be required (Rockström 2002). However, irrigation is too costly make an impact on rural households’ food security for the near future. To reduce the vulnerability to smallholder farmers in semi-arid regions to climate change and variability, and to increase the resilience to climate change there is need to optimize in-field water harvesting techniques so as to improve crop yields. It is therefore imperative to investigate the options to increase water productivity in rain-fed agriculture for increased food production. With improved in-field water harvesting, harvested rainfall can possibly sustain crop production during the mid-season dry spells and this will reduce crop failures and may ultimately lead to improved household food security.

In-situ rain water harvesting, involves the use of methods that increase the amount of water stored in the soil profile by trapping or holding the rain where it falls, and it involves small movements of rainwater as surface runoff, in order to concentrate the water where it is required (UNEP 1997). Water harvesting retains moisture in-situ, through structures that reduce runoff from fields and hold water long enough to allow it to infiltrate. Improved in-field water harvesting can increase the time required for crop moisture stress to set in and thus can result in improved crop yields. Improved water harvesting may result in improved crop yields, food security and livelihood among households. Water harvesting is nothing new but a revival of old techniques that have received little attention since the modernisation of agriculture in the 1940s (Rockström 2002). Water harvesting is believed to have originated from Iraq over 5000 years ago, where methods such as such as diversion of “Wadi” flow onto agricultural fields were used (Hardan 1975; Hatibu and Mahoo 1999). This review will therefore evaluate current in-field water harvesting practices in the smallholder farming areas located in the semi-arid regions of Zimbabwe and other options that can be used for optimising in-field water harvesting to improve the resilience against changing climate.

Zimbabwe is located in southern Africa between 19° and 30° south of the Equator. The country has a total land area of 39 million ha and approximately 21 million ha (54% of the land area) is used for agricultural production. Vincent and Thomas (1961) partitioned the country into five agro-ecological or Natural Regions (NRs), based mainly on the mean annual rainfall (mm year^-1^) which is received between November and April (Table 1). Most cropping is done in NRs I and II (21% of land area), while NRs IV (33%) and V (28%) are considered too risky for crop production. More than one and a half million farming households in the smallholder settlements farm are located on about 49% of the country’s agricultural land, of which >70% is in marginal NRs IV and V. Most of the smallholder farming areas are in the marginal agro-ecological regions which have (i) low rainfall (ii) severe dry spells; and (iii) shallow sandy soils of low fertility (FAO 2006). The data on current water harvesting and conservation practices that have been used and that are currently being promoted were collected from literature that included published and unpublished materials from Government departments in the Ministry of Agriculture such as the Institute of Agricultural Engineering and Agricultural extension services (Agritex), the World Wide Web, non-governmental organization (NGO) and research reports.

**Table 1:** **Natural regions, a real coverage (hectares (ha)) and rainfall distribution in Zimbabwe**

Natural region	Area (000 ha)	% of total land	Annual rainfall (mm)	Farming system
I	613	1.56	>1000	Suitable for dairy farming forestry, tea, coffee, fruit, beef and maize production
II	7 343	18.68	750-1000	Suitable for intensive farming, based on maize, tobacco, cotton and livestock
III	6 855	17.43	650-800	Semi-intensive farming region. Suitable for livestock production, fodder crops and cash crops
IV	13 010 036	33.02	450-650	Suitable for farm systems based on livestock and resistant fodder crops. Forestry, wildlife/tourism
V	10 288	26.2	<450	Extensive farming region. Suitable for extensive cattle Ranching, forestry, wildlife/tourism

## Results and discussion

### Pre-independence soil and water conservation in Zimbabwe

Soil and water conservation in Zimbabwe (formerly Rhodesia) dates back to the early 1900^′^s following the introduction of the plough and permanent settlements. The plough was introduced around 1920^′^s following the arrival of white settlers. The introduction of permanent settlements and the plough also saw the abandonment of traditional farming practices which were used to conserve water in-field. The introduction of the plough was accompanied by massive land degradation and this led to the introduction of the contour ridge that was designed by Alvord in the 1930^′^s (Alvord 1958) and some conservation agriculture practices in the form of Conservation Farming basins that were first implemented in Musana communal lands in the North-eastern part of the country by Brian Oldrieve (Oldrieve 1993).

The contour ridge was mainly targeted for the commercial farming sector in high rainfall areas. However, due to increased land degradation in the newly established smallholder farming areas, contour ridges were also introduced indiscriminately to combat accelerated soil erosion in smallholder farming areas in the 1930s, and later enforced through the Natural Resources Act section 52 in 1941, without considering the rainfall characteristics that had contributed to accelerated erosion after the introduction of the plough in the 1930s (Aylen 1941; Alvord 1958). In semi arid areas contour ridges were inappropriate as they disposed off the precious water from the fields instead of retaining it. Construction of standardized contour ridges has been enforced by governments since the 1930^′^s and due to the enforcement; the whole idea of soil conservation became unpopular among smallholder farmers. The use of contour ridges was resisted by farmers, as it was seen as a tool of oppression because of the brute force used to enforce the law and the high labour demand required for the construction of the contours. Contour ridges took off 15% of the land out of production and there was no appropriate equipment to use. They were also irrelevant to drought prone regions where rainfall is scarce. During the liberation war of the 1970^′^s the concept of “Freedom Farming” was introduced by the freedom fighters and this involved destruction of existing contour ridges as a protest against the colonial regime and this only stopped after independence in 1980. However, to date, the standard contour ridges are to some extent still enforced, but it is now possible to establish other means of soil conservation without actually breaking the law (Dreyer 1997).

### Soil and water conservation efforts of the 1980 and 1990^′^s in Zimbabwe

In 1980, when Zimbabwe became an independent state, the government formulated new policies for the agricultural sector. However, most of these policies failed because the authorities employed a top-down policy, with the government and the secretariat assuming the custodial role (especially regarding the resource-poor farmers). Even today, although perception and approaches are changing, the standard contour ridges are to some extent still predominant, despite the introduction of other practices in soil and water conservation (Gumbo et al. 2012). The uses of water harvesting technologies for improved water use efficiency have been evaluated in several semi arid regions of the country. Farmers in semi arid regions of Chivi have successfully used water harvesting technologies such as the *Fanya ju* and spreading of termitaria (Hagmann and Murwira 1996a) to increase crop yields relative to conventional tillage. In addition, farmers driven innovative soil and water harvesting practices e.g. Infiltration pits (*chibatamvura*), crop rotations, winter cropping, improved tillage techniques and many others have been used in Zimbabwe. A survey by Mutekwa et al. (2005;2006) in ward (Ngundu) of Chivi district in Masvingo with a total population of 9031, showed that infiltration pits, fanya juus, tied ridges, macro-catchments and graded contours were adopted by 61%, 34%, 27%, 10% and 7% of the population respectively.

In the first 20 years after independence efforts to manage water in rain-fed systems using water conservation technologies focused on in-situ water harvesting techniques such as tied ridging (*mariji*), tied furrows and conservation tillage (CONTIL) (Hagmann and Murwira 1996b). The CONTIL project began in Zimbabwe in 1988 to 1996 as a collaborative project between AGRITEX and GTZ implemented with the aim of developing a number of tillage techniques to address problems related to soil loss, water run-off, and declining yields (Vogel 1992). Its initial aim was to reduce soil erosion through improved farmer husbandry techniques and it evaluated three reduced tillage systems (mulch ripping, clean ripping, and tied ridging) against two traditional systems (conventional tillage and hand hoe) (Marongwe et al. 2012). The project evolved in an attempt to promote a completely different way of working within the government extension service. This implied a shift away from Agritex’s rigid, linear, top down extension model, to a more process-oriented approach where farmer driven needs led to the development of the bottom up approach (Hagmann and Murwira 1996b). After five seasons of research, Moyo and Hagmann (1994) concluded that mulch ripping with its higher water-use efficiency appeared to be the most viable conservation tillage treatment in the semi-arid areas of Zimbabwe. However, several on-station and on-farm research activities on conservation tillage and erosion by the Institute of Agricultural Engineering (IAE), Agricultural Research Trust Farm (ART Farm) and Henderson Research Station failed to see any significant uptake of conservation tillage technologies by the smallholder farming sector in Zimbabwe (Marongwe et al. 2012).

Nyagumbo (1999) describes experiences on maize production using tillage systems from on-station and on-farm research in Zimbabwe carried out between 1988 and 1997. On-station, four conservation tillage methods namely no-till tied ridging, mulch ripping, clean ripping and hand hoeing were compared to the control of conventional tillage system and the results showed that on farm maize yields were significantly different from farmer to farmer, depending on their management skills, seasonal rainfall and soil type. The results from the work by Nyagumbo (1999) showed that there was no scope for giving blanket recommendations to farmers on no-till tied ridging. It was also realised from the study that tied ridging alone could not bring better yield results to the farmers; hence there is a need to incorporate soil fertility and other management components for improved productivity. Tied ridges could not work without the support of structures such as contour ridges, infiltration pits and other preventive structures. Tied ridges on sandy soils did not overally increase soil water content within the root zone due to the low water holding capacity of sands.

Despite the effectiveness of some water conservation techniques, adoption by farmers has been poor mainly because of several factors among them; high labour intensity, e.g. in Tanzania, the cost of making tie ridges is estimated at 33% higher than conventional land preparation using hand hoes (Ibraimo and Munguambe 2007). (ii) Available water harvesting techniques have been designed in a “one size fits all” approach as there are no technical guidelines on water harvesting technologies suitable for different climatic and soil conditions. To address these challenges, there is a need of a more efficient capture and use of the scarce water resources in arid and semi-arid areas. An optimization of the rainfall management, through water harvesting in sustainable and integrated production systems can result in improved livelihood of the small-scale farmers’ through improved rain fed agriculture production (Ibraimo and Munguambe 2007).

In 2003, after substantial donor funding targeting improved food security for vulnerable households, there was renewed effort to promote soil and water conservation. Some of the technologies that were promoted included minimum mechanical soil disturbance, maintenance of soil cover with organic materials and diversifying crop rotations or sequencing or associations adapted to local environments (Marongwe et al. 2012). In 2008 the government got involved in conservation agriculture resulting in the launch of the Conservation Agriculture Promotion Network (CAPNET) which brought together different government departments and ministries (Ministry of Agriculture, Mechanization and Irrigation Development 2008) but to date, CAPNET has since been absorbed into the main CA Task Force.

In addition some permanent water conservation practices that were intiated in the 1980s’ such as the zero-grade or dead level contours reinforced with infiltration pits were also promoted by non-governmental organizations (NGOs) in semi arid areas of areas such as Gwanda, Zvishavane and Chiredzi districts during this re-newed effort (Motsi et al. 2004; Mugabe 2004; Munamati and Nyagumbo 2010; Mupangwa et al. 2011; Gumbo et al. 2012). Results from these studies have shown that dead level contours and infiltration pits can contribute towards improved soil water status in the cropped fields, as soil water on both upslope and downslope sides of the infiltration pit was replenished (Mugabe, 2004).

Dead level contours are permanent and they are only made once and they have been reported to increase maize yields in semi arid regions of Zimbabwe (Gumbo et al. 2012). However, though considerable progress has been made with respect to adoption of dead level contours by farmers (Hagmann and Murwira 1996a), little is known about the technical design of these water harvesting techniques for different soils and rainfall regimes. Some of these water harvesting technologies may cause a potential risk of erosion if the quantities of runoff harvested is greater than the capacity of the structures. However, only a few studies e.g. Mupangwa et al. (2006) and Gumbo et al. (2012) have been carried out to assess this.

### Water conservation lessons from other regions

There are several in-field water conservation practices that have been used in several regions of Africa including: earth bunds, planting pits or planting basins and their modifications used in different parts of East and West Africa (Critchley 2009). Planting pit or basin is commonly used in the sub-region with various modifications including the zai pits (*Tassa*) in Burkina Faso, Niger and in Mali, and half moon (*demi-lunes***)** in Niger. The zai pits for concentration of in-field runoff, which originating in Burkina Faso have been practiced for centuries among smallholder farmers in West Africa (Reij et al. 1988). In a study in Niger, Olaleye et al. (2006) reported higher yields on zai treatments compared to flat planting and this was attributed to a build-up in the soil organic matter contents which may have increased the soil water holding capacity in the zai treatments. In Kenya, the zai technique utilizes shallow, wide pits that are about 30 cm in diameter and 15–20 cm in depth, in which four to eight seeds of a cereal crop are planted. In the Njombe district of southern Tanzania, the pits are made bigger and deeper (at least 0.6 m deep), and a 20 liter volume of manure is added and farmers plant about 15 to 20 seeds of maize per pit (Mati 2005).

Another variant of the *zai* is the Chololo pits, a method in which pits with a diameter of 22 cm and a depth of 30 cm are used. The pits run parallel to the contour and have an in-row space of 60 and inter-row spacing of 90 cm. Ashes (to expel termites), farmyard manure and crop residues are added into the pit and this is covered by a little soil, and some space is left to hold runoff water. Some of the soil that was excavated when making the pit is used to make a small bund around the hole. One or two seeds of maize or millet or sorghum are planted per hole and yields have been reported to triple even during very dry years (Mati 2005). A larger version of the zai is the ‘five by nine’ pit, which has surface dimensions of 60 x 60 and a depth of 60 cm. The pits are larger than zai and “five by nine” refers to the five maize seeds (for dry areas) or nine maize seeds (for wet areas) planted at the pit diagonals. This type of pit can be re-used for a period of up to 2 years (Mati 2005).

Micro basins, also called earth basins have also been used in semi-arid regions of Africa to capture and hold rainwater (Kassougue et al. 1996). Micro basins are constructed by making low earth ridges on all sides, they are normally circular, and square or diamond shaped micro-catchments which are 1-2 m in width and about 0.5 m depth (Mati 2005). In addition, earthen bunds which are of various forms of earth-shapings created for ponding runoff water have been used for water harvesting in semi arid regions of Africa (Ibraimo and Munguambe 2007). The variations of earthen bunds include contour bunds, semi-circular bunds and Negarims microcatchments which have been used in arid and semi-arid regions where the seasonal rainfall can be as low as 150 mm (Mati 2005). These earthen bunds have been used widely in Kenya, for example in Busia, district of Kenya, while semi-circular bunds are made by digging out holes along the contours. Negarims microcatchments are regular square earth bunds, which have been turned 45 degrees from the contour to concentrate surface runoff at the lowest corner of the square where there is an infiltration pit dug. Fruit trees can be grown in the pits were all the runoff is concentrated.

Contour earth ridges, which are generally 15–20 cm high, constructed parallel to the contour and spaced 1.5 to 3 m apart, have been found to be useful for producing crops and trees. Contour earth ridges are constructed by digging a furrow along the contour and throwing the soil on the downslope side to form ridges. However, their adoption in Kenya has been limited without technical assistance (Thomas 1997). Broadbed furrow systems are a modification of contour ridges, with a catchment ahead of the furrow and a within-field micro catchment water harvesting systems which are used extensively in Ethiopia, Kenya and Tanzania. The systems are made from small earthen banks with furrows which collect runoff from the catchment area on the higher sides between the ridges. The system is most suitable in areas where the annual rainfall is from 350 mm-700 mm, with even topography, with gentle slopes of about 0.5-3% steepness and soils that are fairly light and have high infiltration rates (Mati 2005). The other water harvesting technologies that have also been successfully used in other countries include the half moon basins in Mali and the low lying crescents in Sudan (El Sammani and Dabloub 1996).

Several recommendations on the different water harvesting technologies have been made from the regions and Zimbabwe can certainly learn from those experiences. For example, in Arusha and Kilimanjaro regions of Tanzania, an evaluation of tied ridges, open ridges, potholes (small holes) and flat planting as techniques for water harvesting showed significant maize yield increases under tie ridging as this method retained more moisture than the other methods. The recommendations were that tied ridges were not suitable where the average annual rainfall is more than 800 mm, as they may cause water logging. In areas with sandy soils, tie ridging is not recommended due to high water percolation and water logging respectively, while in drier areas with about 500 mm rainfall, tie ridging is recommended to farmers who have easy access to capital resources, while potholing is recommended to farmers with scarce resources. Other recommendations included the crest and side seed placement in ridges to eliminate water logging. However, the main problems associated with these water harvesting structures are that they are difficult to construct, have high labour requirements and they do not allow the use of mechanization (Critchley and Siegert 1991). Some of the water harvesting technologies needed to be constructed on an annual basis and this was a reason for poor uptake. From these experiences from the region, it was observed that water harvesting technologies should not be given as a blanket recommendation.

### Future directions of water conservation

Besides the water harvesting technologies that have been promoted in the 1990^′^s, currently some interventions that have been borrowed from other regions of sub Sahara Africa are being promoted or tested in Zimbabwe and these include; basin tillage (*makomba*), a modification of the zai which has been widely promoted under Precision Conservation Agriculture (PCA), half moon basins and shallow planting furrows using a hand hoe among others (Twomlow et al. 2008). These basins were introduced targeting poor and vulnerable households without access to draft power and also during a period when initiatives were promoted by NGOs without the assistance of the government extension system which was largely excluded by the donors. It was only until 2008 that the government got involved in conservation agriculture when they launched the Conservation Agriculture Promotion Network (CAPNET) which has since been absorbed into the national CA Task Force.

In Zimbabwe by the 2007/2008 season, more than 50 000 households had tried the PCA technology and it resulted in increased average cereal yields by 50 to 200% in > 40 000 households (Twomlow et al. 2008). For most of these households, inputs were provided, however, there is need for planning to assure success and sustainability, so that the farmers are able to support themselves without the help of NGOs by the end of the programs. Most of the programs have failed to continue after the NGOs or the Government stopped providing inputs. From the available literature, the major challenges of all the technologies still remain high labour intensity and at times blanket recommendations of interventions and resource constraint of farmers (Munamati and Nyagumbo 2010). For instance *makomba* though they have been adopted by some farmers are still a challenge because of the perennial high labour demand, to the extent that they have been given a nickname, “*diga ufe*” in vernacular large which translate to “*dig and die*”. To date farmers who are willing to adopt such technologies face a lot of stigma from fellow farmers because such technologies are perceived to be for the poor and not for the resource endowed. Resource ownership is also a key factor in farmers’ ability to scale out water harvesting technologies and a study by Munamati and Nyagumbo (2010) showed that performance was significantly linked to resource status.

In the face of these challenges, farmers in semi-arid areas have tended to show more interest in large, semi permanent to permanent water harvesting mechanical structures (Hagmann and Murwira 1996a). In recent years, increased attention has been focused on introducing other options for water harvesting as alternatives to the available technologies. These options include modifications of the standard contour ridges through incorporating infiltration pits (Maseko 1995), deepened contours, fanya juus, tied furrows, the ‘five by nine’ method that is used in Kenya (Mati 2005) and dead level contours (Motsi et al. 2004; Gumbo et al. 2012).

The dead level contour is a farmer driven innovation developed in 1988, which led to the adjustments and modification of the standard graded contours. In 1988, about 10 farmer innovators in Zvishavane and Chivi districts, in Zimbabwe, were part of the Indigenous Soil and Water Conservation in Africa Project to share their knowledge and discuss dead level contour innovations (Hagmann and Murwira 1996a). After that, the technology of dead level contours has spread, and the number of adopters increased to about 5000 in ten pilot districts. However, research has not moved fast enough to scientifically justify the use of these techniques such that little is known about the conditions under which such techniques provide beneficial effects (Nyagumbo et al. 2009b). Some work by NGOs such as Practical Action, have shown that farmers benefit a lot from using dead level contours (Nyagumbo et al. 2009b; Munamati and Nyagumbo 2010; Mupangwa et al. 2011; Gumbo et al. 2012), such that about 40% of the farmers still use these water harvesting strategies although the project that promoted them ended in 2004.

Another water harvesting technology that needs evaluation is the tied contour. To date there are no studies that have evaluated the potential benefits of using the tied contour ridges for water harvesting in semi arid smallholder farming areas of Zimbabwe. The advantage of tied contours is that they are a modification of the standard contour ridges which are already in place in almost every field in the smallholder farming areas. Permanent water harvesting technologies like tied contours are likely to be well received by farmers and future programmes should promote such technologies in addition to current efforts, especially in semi-arid areas. However, the merits of using these technologies are still unknown and this calls for further research to evaluate the performance of tied contours as a water harvesting technology for the semi-arid smallholder farming areas.

Experiences from earlier large scale adoption of soil and water management techniques, for example the wide adoption of soil and water conservation in Machakos district in Kenya (Mati 2005), showed that improved management of soil and water also reduces land degradation. Therefore the adoption of a variety of water conservation technologies which should be availed to farmers in the ‘farmers basket of innovations’ may also control surface runoff and erosion, which currently constitutes the largest source of land degradation in tropical savannahs. There is need to couple soil conservation with other practices with short-term benefits. Research shows smallholder farmers will adopt technologies with short term benefits even if long term benefits are higher.

### Research gaps

Most of the water that is harvested in-field is stored in the soil. In situ water harvesting works better where the soil is deep enough and the water holding capacity is large enough to retain moisture which can be used by the crop during dry spells and where rainfall is equal or more than the crop water requirement. Some of these technologies may not work in areas were the soils are sandy, because of poor water retention. Currently the recommendations for water harvesting technologies give blanket recommendation and do not consider inherent differences in soil water holding capacities, soil depth and texture. Thus, there is need to carry out research on water harvesting across a range of soils so as to recommend the best technology for each soil type. In addition, there is need to integrate water harvesting with improved fertility and crop management in order to increase efficiency of use of the harvested water.

In addition, the effects of combining more than one in-situ water harvesting technology on crop growth are also unknown and this calls for further research. There is also need to identify sustainable mechanisms for promoting evaluated and proven water harvesting techniques as current extension methodologies and donor driven initiatives in Zimbabwe have failed to increase uptake and sustained adoption of such techniques. Exploring farmer-led knowledge sharing platforms should be explored for scaling up proven technologies.

There is need for policies to promote the uptake of in-field water harvesting in semi arid to arid regions of Zimbabwe. Zimbabwe like many other countries in the region has no clear government policies and legislation on the use of in-field water harvesting in semi arid to arid regions (Nyagumbo and Rurinda 2011). The smallholder farmers remain at the “tail-end” of policy making, and in most cases, policies are designed using the top to bottom approach. In addition, there are no incentives that are given to farmers who take up innovative in-field water harvesting. For some farmers the benefits are there but research must clearly show the benefits of adopting improved water harvesting, among them increased yield and improved food security. There are no technical guidelines or manuals that farmers can use as reference material for most water harvesting technologies. In the few cases where the guidelines are available, they are mostly in English and not in a language most farmers would easily understand (Practical Action 2012). Except for the effort by some NGOs, the government has not been active in providing manuals even to its extension staff and the farmers. In addition, the land tenure system in Zimbabwes’ newly established resettlement areas is not well defined. After the fast track land reform that began in 2000, land users in new resettlement areas are not willing to invest in water harvesting technologies, because they lack land tenure security, even though they have the knowledge of the technologies.

There is also need to evaluate promising water conservation strategies from other regions before promoting them in smallholder farming areas in Zimbabwe. A technology may be indigenous to the farming system of origin, while being an innovation to the society of adaptation. Some technologies that have been evaluated in semi arid countries e.g. Kenya and Sudan should be evaluated for suitability to local conditions.

## Conclusions and recommendations

In-field water harvesting is one of the many climate change adaptation strategies that can be adopted by farmers in the semi- arid regions of Zimbabwe. In-field water harvesting can potentially enhance soil water storage, and this will enable crops to survive during mid season droughts. Improved water harvesting may result in improved crop yields, food security and livelihood among households. Water harvesting should be integrated with other management strategies e.g., improving soil fertility management, tillage, timing of operations, pest management and choice of cropping system in order to increase the efficiency of use of the harvested water. Water, is probably the strategic entry point, for reducing risk of crop failure due to water scarcity. However, there is need for changes in policies to promote the use of in-field water harvesting technology in the semi-arid smallholder farming areas, improved extension activities, knowledge dissemination, and the promotion of farmer-led knowledge sharing to increase the resilience of farmers to changing climate. The policies should also address the current land ownership structure to encourage farmers to invest on their land. Water harvesting technologies from other regions also need to be explored and tested in Zimbabwe. More permanent water harvesting technologies may be a solution to the problems of perennial high labour requirements of the current water harvesting practices; hence there is need to promote them. Farmers should be given a ‘farmers basket of innovations’ that is full of options, from which they could select the ones most suited to their complex and diverse agronomic, environmental, climatic, socio-economic conditions and resource endowments.
